# Clinical Outcomes With Notch Inhibitors in Notch‐Activated Recurrent/Metastatic Adenoid Cystic Carcinoma

**DOI:** 10.1002/cam4.70663

**Published:** 2025-03-02

**Authors:** Camilla O. Hoff, Luana G. de Sousa, Flavia Bonini, Eduardo Dal Lago, Kaiwen Wang, Juliana M. Siqueira, Yoshitsugu Mitani, Adel K. El‐Naggar, Renata Ferrarotto

**Affiliations:** ^1^ Department of Thoracic Head and Neck Medical Oncology The University of Texas MD Anderson Cancer Center Houston Texas USA; ^2^ School of Medicine University of Sao Paulo Sao Paulo Brazil; ^3^ Department of Thoracic Imaging The University of Texas MD Anderson Cancer Center Houston Texas USA; ^4^ Division of Pharmacy The University of Texas MD Anderson Cancer Center Houston Texas USA; ^5^ Department of Stomatology, Discipline of Oral and Maxillofacial Pathology, School of Dentistry University of Sao Paulo Sao Paulo Brazil; ^6^ Department of Pathology The University of Texas MD Anderson Cancer Center Houston Texas USA

## Abstract

**Background:**

Adenoid cystic carcinoma (ACC) with NOTCH‐activating mutations presents a clinical challenge due to its poor prognosis. NOTCH inhibitors have emerged as a potential therapy for ACC patients with NOTCH activation. This study aimed to evaluate the efficacy of NOTCH inhibitors in this patient population.

**Methods:**

A retrospective analysis was conducted on patients with metastatic ACC harboring NOTCH pathway activation, who received NOTCH inhibitors at MD Anderson Cancer Center. NOTCH inhibitors included AL101, a gamma‐secretase inhibitor, and brontictuzumab, an antibody targeting NOTCH1. NOTCH pathway activation was assessed through genomic analysis for NOTCH‐activating mutations or immunohistochemistry for NOTCH1 intracellular domain (NICD1). Efficacy endpoints included best overall response (BOR) and progression‐free survival (PFS) per RECIST or MD Anderson bone response criteria.

**Results:**

Twenty‐nine patients were included, with a predominance of solid histology (86%). NOTCH‐activating mutations were identified in 82% of patients, and 95% showed positive NICD1 staining. BOR revealed partial response in 17% of patients, stable disease in 55%, and progressive disease in 28%. Median response duration was longer for AL101 compared to brontictuzumab (9.9 vs. 1.7 months, *p* = 0.04). Median PFS with NOTCH inhibitor was 4.2 months (95% CI 2.7–8.6 months). Progression of nontarget lesions occurred in 34% of patients. Comparison with prior therapy showed longer PFS with NOTCH inhibitors (HR 0.38, 95% CI 0.19–0.78, *p* = 0.0065).

**Conclusion:**

NOTCH inhibitors demonstrate activity in NOTCH‐activated ACC, surpassing the efficacy of observation or prior systemic therapies. However, limited PFS and progression of nontarget lesions suggest the potential need for combination therapy to address ACC heterogeneity.

## 1 Introduction

Adenoid cystic carcinoma (ACC) is a secretory gland malignancy that most often emerges from glands along the upper aerodigestive tract [1]. Despite aggressive curative‐intent therapy for localized primary disease, ACC has a high rate of recurrence and distant metastasis [2, 3]. There is currently no established treatment strategy for recurrent/metastatic disease, representing a significantly unmet need.

Systemic therapy options for recurrent/metastatic ACC are limited, and there is no US Food and Drug Administration (FDA)‐approved systemic agent [4]. Cytotoxic chemotherapy is effective in only 10%–20% of patients [5], with the best results achieved with platinum‐based chemotherapy combination [6]. VEGFR inhibitors can provide prolonged disease stabilization but with low response rates of approximately 10% [5].

Given this context, molecular targeted therapy for recurrent/metastatic ACC remains a highly promising field of research in ref. [7]. ACC has two distinct subtypes with drastically distinct prognoses and different targetable alterations [8]. ACC‐I is aggressive, with a median overall survival (OS) of 3 years, whereas ACC‐II is more indolent, with a median OS of 23 years [8]. Genotypically, the aggressive form of ACC is enriched for NOTCH‐activating mutations, particularly in NOTCH1 which are present in approximately 25% of cases and are significantly associated with shorter OS, higher likelihood of solid histology, higher rate of bone and liver metastases, and shorter relapse‐free survival [9, 10, 11]. Mutations in NOTCH 2, 3, and 4 occur less frequently (2%–3%) and its biological role is not well established, however, it also preferentially occurs in the ACC‐I subtype associated with a more aggressive disease phenotype [8, 9, 10].

**FIGURE 1:**
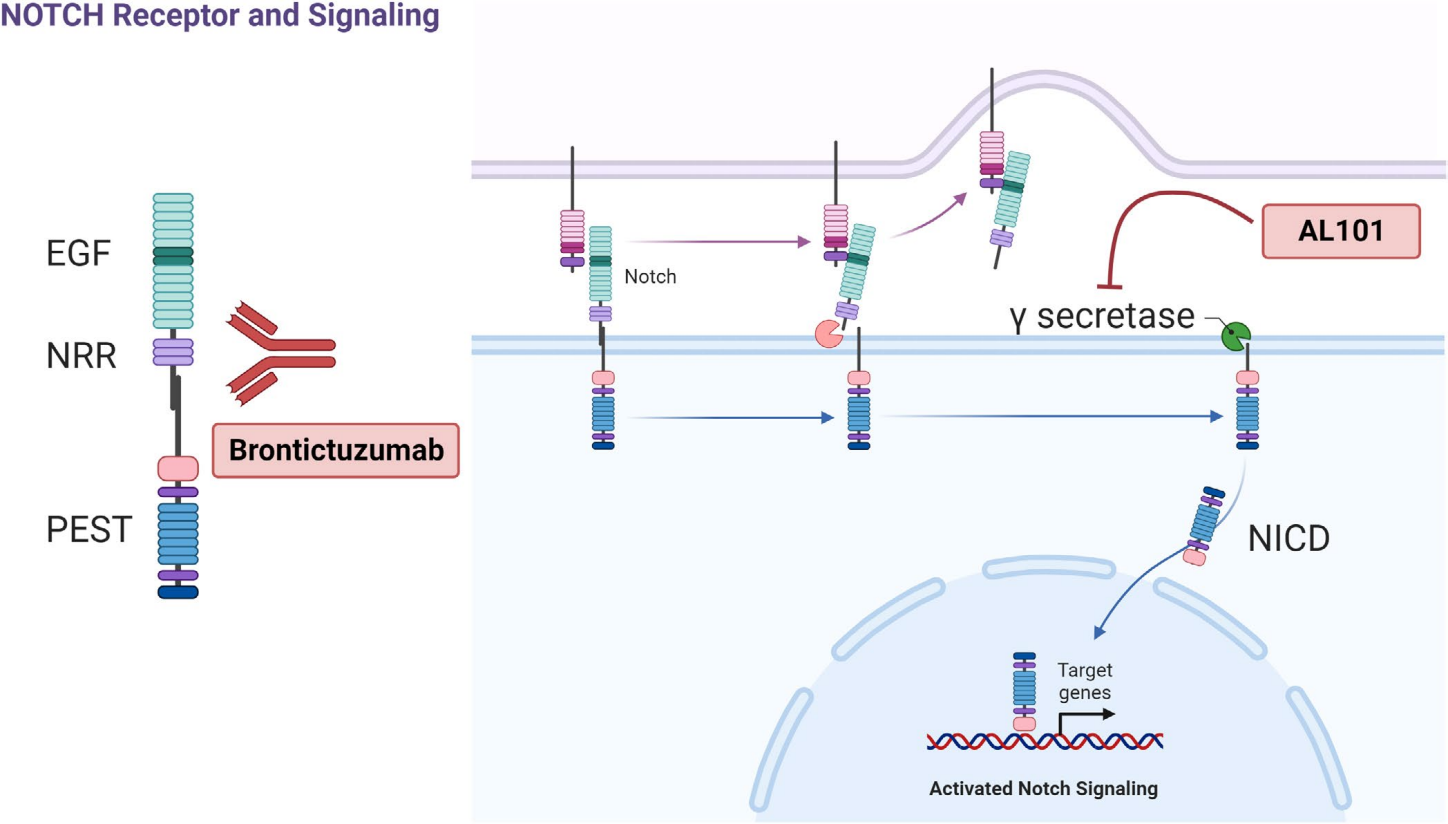
NOTCH signaling pathway and NOTCH inhibitor targets. NOTCH is a transmembrane receptor activated by cleavage by intracellular gamma‐secretase. Following cleavage, the released NOTCH intracellular domain (NICD) translocates to the nucleus and modulates transcription. The monoclonal antibody brontictuzumab targets NOTCH1, inhibiting pathway activation. AL101 inhibits NOTCH activation by inhibiting gamma secretase, disrupting intracellular release of NICD. EGF, epidermal growth factor repeats; NRR, negative regulatory region; PEST, PEST domain.

The discovery that NOTCH1 pathway activation is associated with aggressive ACC led to clinical trials of NOTCH inhibitors in NOTCH‐activated ACC [12, 13, 14]. NOTCH is a transmembrane receptor with extracellular epidermal growth factor (EGF)‐like repeats (Figure 1) [15]. Following ligand binding, the receptor is cleaved by intracellular gamma‐secretase, which releases the NOTCH intracellular domain (NICD), which translocates to the nucleus and activates transcription of target genes [15]. Brontictuzumab is a monoclonal antibody that targets NOTCH1, inhibiting pathway activation (Figure 1). In a phase I clinical trial of brontictuzumab in patients with multiple types of solid tumors, ACC patients had the best efficacy results, with a 17% (2/12) partial response (PR) rate and a 25% (3/12) rate of stable disease (SD) for over 6 months [12]. AL101 is a gamma‐secretase inhibitor that blocks NOTCH signaling (Figure 1); in a phase II trial with 82 patients with *NOTCH*‐mutated recurrent/metastatic ACC, AL101 produced a 69% disease control (PR plus SD) rate, and 12% of patients had a PR [13].

Given that both trials required evidence of NOTCH pathway activation, we hypothesized that these ACC cohorts were homogeneous for particularly aggressive forms of ACC. Therefore, we conducted this retrospective study of patients with ACC treated with NOTCH inhibitors in these two clinical trials at our center to fully contextualize efficacy results given patient history and to compare the efficacy of NOTCH inhibitors with the efficacy of the patients' previous therapy.

## 2 Materials and Methods

### 2.1 Patient Selection and Data Collection

This was a retrospective study, reviewed and approved by the Institutional Review Board of MD Anderson Cancer Center. Electronic medical records were searched for patients with ACC treated at MD Anderson on either the phase I clinical trial of brontictuzumab (NCT01778439) or the phase II clinical trial of AL101 (NCT03691207). Brontictuzumab patients were treated at a dose of 1.5 mg/kg every 3 weeks. AL101 patients were treated at a dose of 4 or 6 mg/kg every week. All patients treated at MD Anderson on either study were included in this analysis.

Clinical data collected included diagnosis date, primary tumor site, ACC histologic subtype, TNM stage at diagnosis according to the *AJCC Cancer Staging Manual*, seventh edition [16], date of diagnosis and site of metastases, *NOTCH* mutation status, NOTCH1 intracellular domain (NICD1) immunohistochemistry (IHC) results, p63 IHC results, best overall response (BOR) to therapy, date of progression on therapy, and follow‐up and survival information. Solid histologic subtype (“histology”) was defined as the presence of any solid component in the tumor tissue. Basaloid histology, a subtype of solid ACC, was defined as basaloid cytologic appearance, with rounded nests and trabeculae, with a fibromyxoid stroma [17, 18]. NICD1 IHC was performed and results were scored in accordance with the brontictuzumab clinical trial protocol [12]. P63 IHC results were scored in accordance with our previously published binary score for ACC subtypes [8].

### 2.2 Statistical Analysis

Patient demographics, disease characteristics, and clinical outcomes were summarized using descriptive statistics. Efficacy endpoints were BOR to therapy according to RECIST 1.1 or the MD Anderson bone response criteria (for patients with bone disease only) and progression‐free survival (PFS) per RECIST 1.1 [19]. Efficacy endpoints were assessed during two periods: during NOTCH inhibitor therapy and immediately before NOTCH inhibitor therapy, during prior systemic therapy or on observation for the same patients.

The Kaplan–Meier method was used for time‐to‐event analysis, including PFS before and during NOTCH inhibitor therapy and OS from diagnosis. Median time to event in months or years with a 95% CI was calculated. A log‐rank test was used to evaluate the difference in time‐to‐event endpoints between patient groups. Alive patients were censored at the last follow‐up date. A *p*‐value of < 0.05 was defined as statistically significant.

Statistical analyses were performed with R Statistical software (version 2.14.0; R Foundation for Statistical Computing, Vienna, Austria).

## 3 Results

### 3.1 Patient and Disease Characteristics

A total of 29 patients with recurrent/metastatic ACC treated with NOTCH inhibitors were included in this analysis. Baseline clinicopathologic characteristics are summarized in Table 1. The median age was 45 years (range: 26–72 years), and 18 patients (62%) were male. All patients had evidence of NOTCH pathway activation, either NOTCH‐activating mutation or NICD1 overexpression by IHC. Twenty‐eight patients had genotyped tumors; of these, 23 (82%) had NOTCH‐activating mutations, of whom 5 had multiple *NOTCH1* mutations. One patient had a NOTCH2 mutation and no *NOTCH1* mutation. The most common site of *NOTCH1* mutation was the PEST domain, followed by the negative regulatory region (NRR) (Figure S1). Twenty patients had IHC for NICD1, and 19 (95%) had positive results. The details of NOTCH activation for each patient are found in Table 2.

**TABLE 1:** Baseline patient characteristics (*N* = 29).

	No. (%)
Age at diagnosis, years	
Median (range)	45 (27–72)
Sex	
Male	18 (62)
Female	11 (38)
M category at diagnosis	
M0	13 (45)
M1	10 (34)
Unknown	6 (21)
Primary tumor site	
Major salivary glands	10 (34)
Minor salivary glands	11 (38)
Other glands	7 (24)
Unknown	1 (3)
Histology	
Solid	25 (86)
Nonsolid	4 (14)
NOTCH mutation status	
Mutant	23 (79)
Wild‐type	5 (17)
Tumor not genotyped	1 (3)
NICD1 IHC results	
Positive	19 (66)
Negative	1 (3)
IHC not performed	9 (31)
P63 IHC results	
Positive	1 (3)
Negative	21 (72)
Unknown	7 (24)
Metastatic disease	
Yes	28 (97)
No	1 (3)
Time to metastasis (*N* = 28)	
At diagnosis	10 (36)
< 3 years	12 (43)
≥ 3 years	6 (21)
Number of sites of metastasis (*N* = 28)	
One	6 (21)
Two	7 (25)
Three or more	15 (54)
Sites of metastasis (*N* = 28)	
Lung	20 (71)
Bone	20 (71)
Liver	17 (61)
Pleura	8 (29)
Lymph node	4 (14)
Brain	4 (14)
Number of systemic therapy lines before NOTCH inhibitor therapy	
0	11 (38)
1	11 (38)
≥ 2	7 (24)
Systemic therapy immediately before NOTCH inhibitor therapy (*N* = 18)	
Chemotherapy	7 (39)
EGFR inhibitor	2 (11)
VEGFR inhibitor	1 (6)
Immunotherapy	1 (6)
Others as part of clinical trial	7 (39)

Abbreviations: IHC, immunohistochemistry; NICD1, NOTCH1 intracellular domain.

**TABLE 2:** Treatment, NOTCH activation, and outcome data for individual patients.

Patient ID	NOTCH inhibitor	Histology	P63 status	NICD1 IHC status	NOTCH‐activating mutation status	Number of NOTCH mutations	NOTCH mutation location(s)	BOR to NOTCH inhibitor	PFS, mo
1	AL101	Solid	Unknown	Positive	Mut	2	PEST	SD	4.2
2	AL101	Solid	Negative	Positive	Mut	NK	NK	PR	6.8
3	AL101	Solid	Negative	Positive	Mut	2	PEST; EGF	PD	2.0
4	AL101	Solid	Negative	Positive	Mut	1	NRR	SD	3.7
5	AL101	Solid	Unknown	Positive	Mut	1	PEST	PD	1.0
6	AL101	Solid	Negative	Positive	Mut	1	NRR	SD	4.2
7	AL101	Solid	Unknown	Positive	Mut	2	PEST; EGF	SD	4.7
8	AL101	Solid	Negative	Positive	Mut	3 (1 N2)	PEST; *NOTCH2*	SD	8.9
9	AL101	Solid	Negative	NK	Mut	NK	NK	PR	9.9
10	AL101	Solid	Negative	NK	Mut	1	PEST	SD	7.4
11	AL101	Solid	Unknown	NK	Mut	1	PEST	SD	9.3
12	AL101	Solid	Negative	NK	Mut	1	PEST	PR	11.2
13	AL101	Solid	Negative	NK	Mut	1	PEST	SD	2.7
14	AL101	Solid	Negative	Equivocal	Mut	1	NRR	SD	6.5
15	AL101	Solid	Negative	Negative	Mut	1 (N2)	*NOTCH2*	PD	0.4
16	AL101	Nonsolid	Negative	NK	Mut	3	PEST; EGF	SD	3.7
17	AL101	Solid (Basaloid)	Negative	NK	Mut	1	NRR	SD	4.1
18	AL101	Solid (Basaloid)	Negative	NK	Mut	1	PEST	PD	1.9
19	Brontic	Solid (Basaloid)	Negative	Positive	Mut	2	PEST; NRR	PR	2.3
20	Brontic	Solid	Negative	Positive	Mut	NK	NK	PD	1.9
21	Brontic	Solid	Negative	Positive	Mut	1	NRR	PD	1.7
22	Brontic	Solid	Negative	Positive	Mut	2	PEST; NRR	PD	1.4
23	Brontic	Solid	Negative	Positive	Mut	1	PEST	SD	2.9
24	Brontic	Solid	Negative	Positive	WT	NA	NA	SD	8.6
25	Brontic	Solid	Unknown	Positive	WT	NA	NA	PR	1.2
26	Brontic	Nonsolid	Unknown	Positive	WT	NA	NA	SD	8.1
27	Brontic	Nonsolid	Unknown	Positive	WT	NA	NA	SD	12.2
28	Brontic	Nonsolid	Positive	Positive	WT	NA	NA	SD	2.7
29	Brontic	Solid	Negative	Positive	NK	NA	NA	PD	0.4

Abbreviations: BOR, best overall response per RECIST; Brontic, brontictuzumab; EGF, epidermal growth factor repeats; IHC, immunohistochemistry; Mut, mutant; N2, *NOTCH2*; NA, not applicable; NICD1, NOTCH1 intracellular domain; NK, not known; NRR, negative regulatory region; PEST, PEST domain; PFS, progression‐free survival; WT, wild‐type.

Of the 23 patients with known stage at diagnosis, 10 (43%) had metastasis at diagnosis. ACC histology was predominantly solid (25/29, 86%). Twenty‐eight patients (97%) received NOTCH inhibitor therapy for metastatic disease, and 22 of these 28 (79%) presented with 2 or more sites of metastasis. The most common sites of metastasis were bone (20/28; 71%), lung (20/28; 71%), and liver (17/28; 61%). Of all 29 patients, 18 (62%) received systemic therapy prior to NOTCH inhibitor, with seven patients (39%) having received cytotoxic chemotherapy (Table 1).

### 3.2 Median OS

The median OS from diagnosis for the patients in the analysis was 3.1 years (95% CI 2.5–5.7 years). Patients with nonsolid histology had significantly longer median OS than patients with solid histology (238 months [95% CI 68 months to not reached (NR)] vs. 32 months [95% CI 25–45 months]; *p* = 0.044; Figure S2a). Patients with *NOTCH* mutations had significantly shorter median OS than patients with wild‐type *NOTCH* (33 months [95% CI 25–45 months] vs. 87 months [95% CI 68 months to NR]; *p* = 0.036; Figure S2b).

### 3.3 Efficacy of NOTCH Inhibitors

**FIGURE 2:**
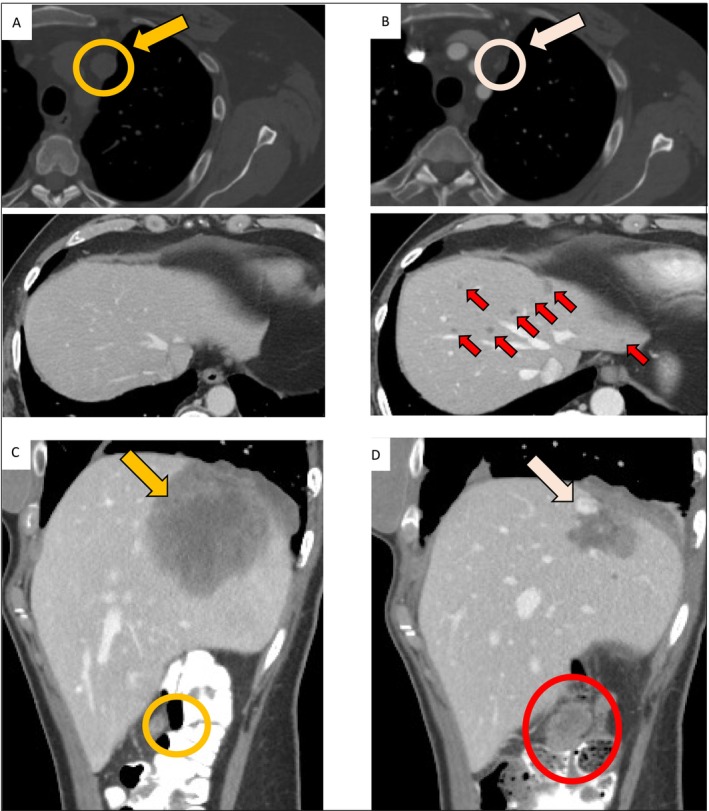
Representative images of mixed responses to NOTCH inhibitor. (A, B) Computerized tomography (CT) of the chest (top) and abdomen (bottom) at baseline (A; yellow circle and arrow) and at the second re‐staging at 16 weeks (B) demonstrates response in the mediastinal nodal metastasis (white circles and arrows) but development of new liver metastases (red arrows). (C, D) CT of the abdomen in another NOTCH inhibitor trial patient at baseline (C; yellow circle and arrow) and at second re‐staging at 16 weeks (D) demonstrates response in a hepatic lesion (white arrows), but progression of a peritoneal metastasis (red circles).

Of the 29 included patients, 18 (62%) received AL101, and 11 (38%) received brontictuzumab. NOTCH inhibitor was given in the second line or beyond in 18 patients (62%). The efficacy outcomes are summarized in Table 3. The overall rate of response (ORR) to NOTCH inhibitors was 17% (5/29; 95% CI 6–36), and all five responses were PRs. Of these 5 PRs, 3 were in patients receiving AL101, and 2 were in patients receiving brontictuzumab, for ORRs of 17% (3/18; 95% CI 4–41) and 18% (2/11; 95% CI 2–52), respectively. Notably, only the three PRs on AL101 were confirmed on a subsequent scan; all two PRs on brontictuzumab were unconfirmed. Consistently, median duration of response was 9.9 months (95% CI 6.8 months to NR) for AL101, compared to 1.7 months (95% CI 1.2 months to NR) for brontictuzumab (*p* = 0.04; Figure S3). All five patients with PR had solid histology, and 4 of the 5 had *NOTCH* mutations (Table 2). Of note, the 5 patients who had a PR to a NOTCH inhibitor had PD as BOR on their previous therapy. SD was the most frequent BOR to both NOTCH inhibitors, occurring in 11 patients (61% [95% CI 36–83]) receiving AL101 and 5 (45% [95% CI 17–77]) receiving brontictuzumab. Of note, ACC patients treated with NOTCH inhibitor had a high rate of mixed responses: 10 of the 29 patients (34%) experienced progression of nontarget lesions (Figure 2).

**TABLE 3:** Efficacy of NOTCH inhibitors.

Efficacy endpoint	NOTCH inhibitors (*N* = 29)	AL101 (*N* = 18)	Brontictuzumab (*N* = 11)
Best overall response, no. (%)			
Complete response	0 (0)	0 (0)	0 (0)
Partial response	5 (17)	3 (17)	2 (18)
Stable disease	16 (55)	11 (61)	5 (45)
Progressive disease	8 (28)	4 (22)	4 (36)
Time‐to‐event endpoints, mo			
Median PFS (95% CI)	4.2 (2.7–8.6)	4.7 (3.7–NR)	2.7 (1.7–NR)
Median duration of response (95% CI)	6.8 (NR–NR)	9.9 (NR–NR)	1.7 (NR–NR)

Abbreviations: NR, not reached; PFS, progression‐free survival.

**FIGURE 3:**
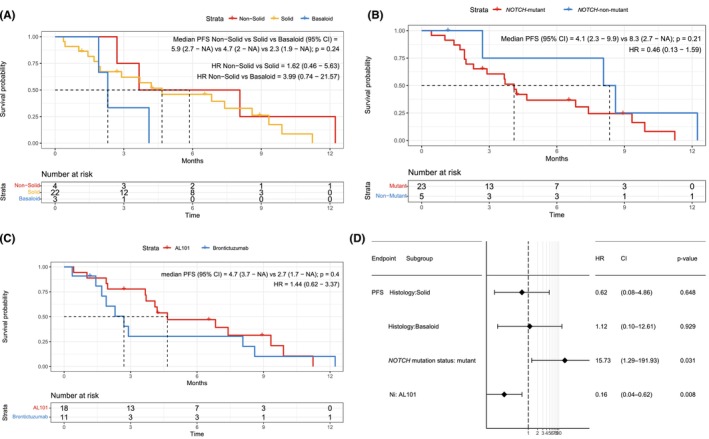
Univariate and multivariate analyses of PFS on NOTCH inhibitor. (A) Nonsolid histology was associated with longer PFS than solid histology. (B) NOTCH‐wild‐type ACC was associated with longer PFS than NOTCH‐mutant ACC. (C) AL101 was associated with numerically longer PFS than brontictuzumab, but this difference was not significant on univariate analysis. (D) On multivariate analysis, AL101 was associated with longer PFS than brontictuzumab. HR, hazard ratio; Ni, NOTCH inhibitor.

The median PFS on NOTCH inhibitor was 4.2 months (95% CI 2.7–8.6 months). When we compared PFS by histology, a known prognostic factor [20], we observed longer PFS for nonsolid than for solid or basaloid histology (median PFS, 5.9 vs. 4.7 vs. 2.3 months, respectively, *p* = 0.24; Figure 3A). Patients with wild‐type *NOTCH* had longer PFS than patients with mutant *NOTCH*, although this was not significant on univariate analysis (8.3 vs. 4.1 months, *p* = 0.21; Figure 3B). Given that the *NOTCH*‐mutant patients had mutations in different domains of the NOTCH receptor, we compared PFS between patients with mutations in the PEST domain and NRR. We found no significant difference between the groups (Figure S4). The 1 patient with only a NOTCH2‐activating mutation (patient 15 in Table 2) had a very low PFS of 0.4 months.

On univariate analysis, there was no significant difference in PFS between AL101 and brontictuzumab (Figure 3C), although median PFS on AL101 was numerically longer (4.7 vs. 2.7 months). Considering that all patients with wild‐type *NOTCH* and 75% of the patients with nonsolid histology were in the brontictuzumab trial, we performed a multivariate analysis including known ACC prognostic factors, such as NOTCH mutation status and histology, to assess how both these confounding variables impacted PFS for each NOTCH inhibitor. On multivariate analysis, PFS was significantly longer with AL101 than with brontictuzumab (*p* = 0.008; Figure 3D).

One patient in the AL101 trial had repeated circulating tumor DNA (ctDNA) analyses after diagnosis of ACC (Figure S5). Her *NOTCH1* mutational variant was detectable in plasma. While in the trial, the patient experienced tumor shrinkage with BOR of SD, which was reflected in falling levels of ctDNA. The CtDNA levels increase shortly before radiological detection of progression.

### 3.4 Efficacy of NOTCH Inhibitors Compared With Efficacy of Prior Systemic Therapy or Observation

Twenty‐three patients (79%) were eligible for RECIST analysis immediately before NOTCH inhibitor therapy. Thirteen (57%) received systemic therapy before NOTCH inhibitor therapy, while 10 (43%) were under close observation. Of the 13 patients who received systemic therapy before NOTCH inhibitor therapy, 4 (31%) were receiving cytotoxic chemotherapy, and 7 (54%) were in clinical trials. Of the 13 patients, none had a response to pre–NOTCH inhibitor therapy, and 1 (in a clinical trial) had SD, for a clinical benefit rate of 8% (Table 4). All 10 patients under observation had progression per RECIST before NOTCH inhibitor therapy.

**TABLE 4:** Comparison of efficacy between NOTCH inhibitors and regimen immediately before NOTCH inhibitor therapy.

Efficacy endpoint	NOTCH inhibitor (*N* = 29)	Systemic therapy before NOTCH inhibitor (*N* = 13)	Observation before NOTCH inhibitor (*N* = 10)
Best overall response, no. (%)			
Complete response	0 (0)	0 (0)	0 (0)
Partial response	5 (17)	0 (0)	0 (0)
Stable disease	16 (55)	1 (68)	0 (0)
Progressive disease	8 (28)	12 (92)	10 (100)
Time‐to‐event endpoint, mo			
Median PFS (95% CI)	4.2 (2.7–8.6)	2.2 (1.9–NR)	3.6 (2.9–NR)

Abbreviations: NR, not reached; PFS, progression‐free survival.

**FIGURE 4:**
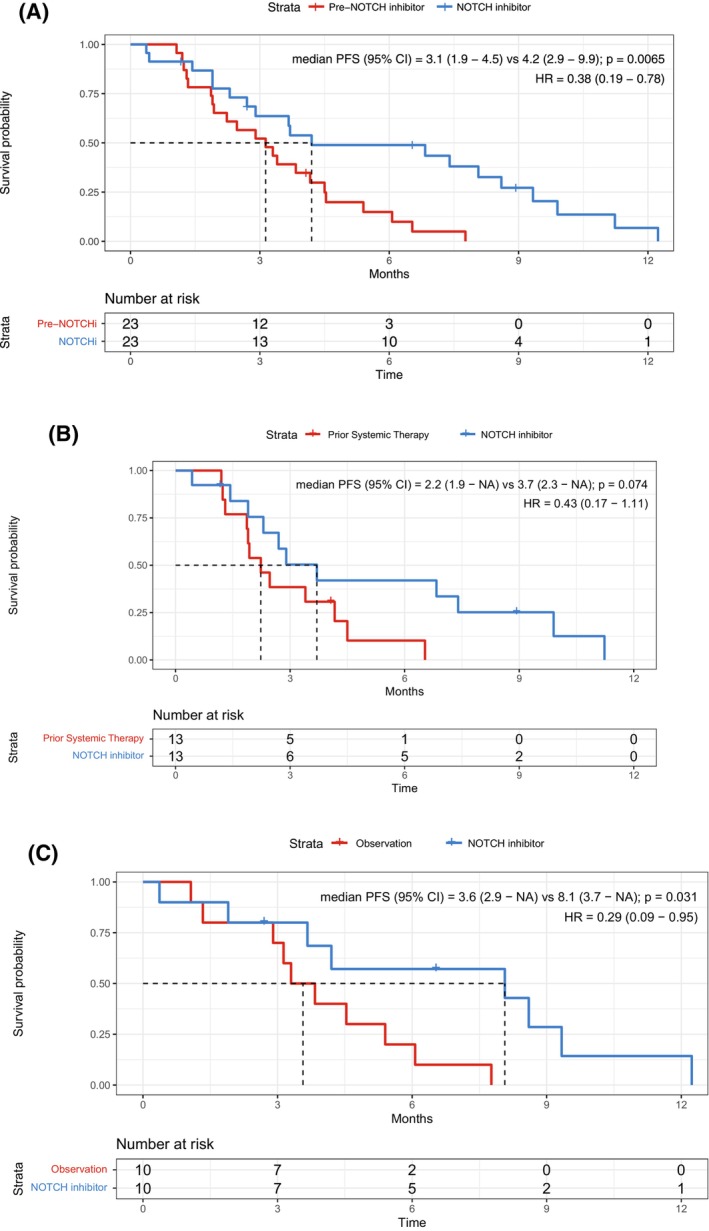
Efficacy of NOTCH inhibitor compares favorably to the efficacy of pre‐NOTCH‐inhibitor systemic therapy or observation. (A) For the 23 patients eligible for PFS analysis before NOTCH inhibitor therapy, NOTCH inhibitor significantly increased PFS when compared to immediately before NOTCH inhibitor therapy, including patients receiving prior systemic therapy or observation. (B) For the 13 patients receiving a prior systemic therapy, median PFS on NOTCH inhibitor was numerically longer than on prior systemic therapy. (C) For the 10 patients under observation before NOTCH inhibitor therapy, median PFS on NOTCH inhibitor was statistically significantly longer than on prior observation. HR, hazard ratio.

For the 23 patients eligible for PFS analysis before NOTCH inhibitor therapy, we compared PFS during the regimen before NOTCH inhibitor therapy with PFS during NOTCH inhibitor therapy (Table 4). Overall, PFS was significantly longer with NOTCH inhibitors than with the prior therapy (HR 0.38 [95% CI 0.19–0.78], *p* = 0.0065, Figure 4A). In the 13 patients who received systemic therapy before NOTCH inhibitor therapy, PFS was longer with NOTCH inhibitors than with the prior therapy (HR 0.43 [95% CI 0.17–1.11], *p* = 0.074, Figure 4B), although the difference was not statistically significant, likely due to the small sample size. In the 10 patients under observation before NOTCH inhibitor therapy, PFS was significantly longer with NOTCH inhibitors than with observation (HR 0.29 [95% CI 0.09–0.95], *p* = 0.031, Figure 4C).

## 4 Discussion

In this study, we aimed to fully characterize the ACC patient cohorts treated with the NOTCH inhibitors AL101 and brontictuzumab at our center and explore the efficacy of these drugs within the context of the patients' prior therapy. As expected, our cohort of patients with NOTCH‐activated ACC presented with features of aggressive disease. The median OS from diagnosis was 3.1 years, in line with reported survival data on NOTCH‐activated ACC [8]. In our cohort, 43% of patients had metastatic disease at diagnosis, compared to the estimated 12% in ACC overall [21]. Of the 28 patients with metastasis at the time of treatment, 22 (79%) presented with multiple sites of metastases, a percentage much higher than in previously reported ACC cohorts [22]. Additionally, the frequency of bone and liver metastases in our cohort was higher than that previously reported in ACC patients [22], with bone disease occurring at the same frequency as lung disease.

Eighty‐six percent of the patients in our cohort had solid histology, a well‐established negative prognostic factor that has been associated with NOTCH‐activating mutations [9, 10, 23]. Basaloid histology, seen in three of our patients, is considered a rare histologic subtype of solid ACC, also associated with aggressive disease and presence of NOTCH1 mutations, although it is unclear how solid‐basaloid subtype compares with other solid ACC [17, 24]. Consistently, in our cohort, solid histology was significantly associated with worse OS (Figure S2a).

Both the AL101 and brontictuzumab trials required evidence of NOTCH pathway activation for enrollment, but the trials used different methodologies to determine NOTCH pathway activation status. For the AL101 trial, patient tumors were assessed for known NOTCH‐activating mutations in the PEST and/or NRR. In our analysis, we found no significant difference in PFS between patients with PEST domain mutations and NRR domain mutations (Figure S4).

For the brontictuzumab trial, NOTCH pathway activation was assessed through IHC staining for NICD1 [12, 25]. Consistently, as NICD1 only detects cleaved NOTCH1, our patient with NOTCH2‐activating mutation on the AL101 trial was the only known NICD1‐negative patient. NICD1 IHC is a highly sensitive assay, and results can be positive even in the absence of NOTCH‐activating mutation, as seen in our cohort [12, 25]. However, when NICD1 staining is seen in a diffuse pattern with solid histology, the frequency of NOTCH1‐activating mutations increases [26]. Our results were consistent with these previously reported observations: all three patients with NICD1‐positive ACC with wild‐type *NOTCH* had nonsolid histology.

Our multivariate analysis revealed significantly worse PFS in patients with mutant *NOTCH* than in those with wild‐type *NOTCH* (*p* = 0.031; Figure 3D). Previous preclinical data on AL101 in ACC patient‐derived xenograft models showed significant response to NOTCH inhibitor in *NOTCH*‐mutant models, but not in *NOTCH*‐wild‐type models [27]. This suggests that the increased PFS we saw in our patients with wild‐type NOTCH was due to more indolent ACC, a hypothesis supported by the high frequency of nonsolid histology (Table 2) and significantly increased OS (Figure S2b; *p* = 0.036) in the patients with wild‐type *NOTCH* [28]. Additionally, of the five patients with PR to NOTCH inhibitor, 4 (80%) had mutant *NOTCH*. The 1 patient with PR and wild‐type NOTCH (patient 25 in Table 2) had the lowest duration of response: 1.2 months.

In our cohort, AL101 was more effective than brontictuzumab. The ORRs were similar for AL101 (17%) and brontictuzumab (18%), but only responses to AL101 were confirmed on a subsequent scan. The median duration of response was 9.9 months for AL101, compared to 1.7 months for brontictuzumab (*p* = 0.04). For both drugs, SD was the most common BOR (AL101, 61%; brontictuzumab, 45%). AL101 was associated with significantly longer PFS than brontictuzumab in a multivariate analysis that controlled for the fact that the brontictuzumab trial included higher proportions of patients with nonsolid histology and wild‐type *NOTCH* (Figure 3D, *p* = 0.008). The distinct mechanisms of action and differences in patient selection likely explain the difference in efficacy seen between Notch inhibitors. AL101 inhibits gamma‐secretase‐mediated NOTCH1‐4 signaling while Brontictuzumab is a monoclonal antibody that inhibits NOTCH1 receptor binding to its ligands (Figure 1), which could lead to limited activity in the presence of NOTCH mutations that leads to ligand‐independent receptor‐cleavage. Furthermore, patients were selected for brontictuzumab treatment based on NICD1 expression by IHC, while all patients treated with AL101 had confirmed NOTCH mutations predicted to be activating [12, 13, 27].

Despite promising preclinical data with gamma‐secretase inhibitors such as AL101, clinical results with gamma‐secretase inhibitors in various malignancies have been below expectations [29], with the notable exception of results in desmoid tumors [30]. One proposed limitation of these drugs is the impact of inhibiting gamma‐secretase on other cellular pathways, especially for immune cells [29]. Inhibition of gamma‐secretase has been associated with increased T‐regulatory cell function and decreased CD8+ cytotoxic T‐cell activity, both potentially decreasing antitumor immunity [29, 31, 32, 33]. An additional concern with gamma‐secretase inhibitors is the potential for gastrointestinal toxic effects [34]; however, preliminary results from the AL101 ACCURACY trial indicate that grade 3 or higher gastrointestinal adverse events occurred at a rate of 10% or less [13]. A third NOTCH‐targeting strategy recently proposed is to target the NOTCH pathway at the transcriptional level [34]. CB‐103 acts by selectively blocking interaction of NICD with the NOTCH transcriptional complex, inducing transcriptional downregulation of the NOTCH pathway [34]. While preclinical results with CB‐103 were promising, a phase I study in ACC produced no objective responses and produced a rate of SD similar to that seen with AL101: 58% [14]. Of note, this trial did not require evidence of NOTCH activation, which may have led to a more indolent cohort [14].

The efficacy of NOTCH inhibitors in our patient cohort compares favorably with the efficacy of existing systemic therapies for ACC. Current treatment options for recurrent/metastatic ACC are limited, with no FDA‐approved systemic therapy and no consensus on when to start therapy [4]. ORRs for single‐agent cytotoxic chemotherapy have been approximately 12%, and ORRs for combination chemotherapy have been approximately 15%–20% [35, 36, 37, 38, 39]. VEGFR inhibitors are a potential systemic therapy alternative for recurrent/metastatic ACC [40, 41, 42, 43, 44, 45, 46]; however, while VEGFR inhibitors produce high disease stabilization rates, the ORR remain close to 6% [5, 47]. In our study, NOTCH inhibitor therapy provided a clear advantage over prior systemic therapy in terms of BOR, with an ORR of 17% for NOTCH inhibitors versus 0% for the prior systemic therapy (Table 4). NOTCH inhibitors were also associated with significantly longer PFS when compared to prior systemic therapy or observation (Figure 4A, *p* = 0.0065). The NOTCH inhibitor ORR of 17% is comparable to ORR seen with combination chemotherapy, widely considered to be the treatment modality that offers the largest probability of major shrinkage [5]. Additionally, this ORR was especially encouraging considering that the NOTCH inhibitor trials selected for ACC patients with NOTCH activation, which is associated with particularly aggressive disease [9], and considering that our patient cohort was heavily pretreated, with 62% of patients receiving NOTCH inhibitor in the second line or beyond.

This analysis of NOTCH inhibitor‐treated patients also revealed some of the major challenges to incorporating NOTCH inhibitors into clinical management of ACC. The high rate of mixed responses to therapy (34%) and the overall limited PFS (4.2 months) strongly favor the investigation of combinations of NOTCH inhibitors with other agents. ACCs have a biphasic cellular composition, often with multiple histologic subtypes represented [23, 48, 49]. A single‐cell RNA analysis found that several genes were differentially expressed in primary tumors and local recurrences or metastases, with the local recurrences exhibiting increased NOTCH signaling [49]. While the high heterogeneity of ACC tumors and metastases is still underexplored, it may explain the high rate of mixed response and relatively low PFS we observed. Combination therapy may enhance NOTCH inhibitor activity and has been demonstrated preclinically using ACC patient‐derived xenograft models [50]. NOTCH signaling is more involved in tumor growth than survival, especially through *MYC* pathway activation, which favors a cytostatic as opposed to cytotoxic effect of NOTCH inhibition [51]. Combinations of NOTCH inhibitors with more cytotoxic agents may increase rates of response. Additionally, NOTCH pathway activation has been implicated in tumor drug resistance through microRNA regulation, formation of cancer stem cells, and epithelial‐to‐mesenchymal transition [52, 53, 54, 55]. Given ACC's low response rates to chemotherapy and other systemic therapies, combinations of NOTCH inhibitors with other agents may reduce intrinsic drug resistance.

Our study has limitations. The retrospective design precludes any definitive conclusions about how NOTCH inhibitors compare with other systemic therapies and observation. However, we conducted a matched‐pair comparative analysis to minimize interference of potential confounding variables. An additional limitation is the small sample size. The objective of our study was to conduct an exploratory analysis of the impact of NOTCH inhibitors on patients with particularly aggressive NOTCH‐activated ACC. The findings here support further exploring NOTCH as a therapeutic target in ACC.

## 5 Conclusions

NOTCH inhibitors have activity in ACC patients with NOTCH‐activating mutations or NOTCH pathway activation. The efficacy of NOTCH inhibitors compares favorably with the efficacy of systemic therapies administered and observation conducted before NOTCH inhibitor therapy. The gamma‐secretase inhibitor AL101 was more effective than brontictuzumab. The limited PFS and high rate of progression of nontarget lesions suggest that NOTCH inhibitor combination therapy may be necessary to address ACC intratumoral heterogeneity.

## Author Contributions

**Camilla O. Hoff:** data curation (equal), formal analysis (equal), investigation (equal), validation (equal), writing – original draft (equal), writing – review and editing (equal). **Luana G. de Sousa:** data curation (equal), investigation (supporting), writing – review and editing (supporting). **Flavia Bonini:** data curation (equal), writing – review and editing (supporting). **Eduardo Dal Lago:** data curation (equal), writing – review and editing (supporting). **Kaiwen Wang:** data curation (equal), writing – review and editing (supporting). **Juliana M. Siqueira:** data curation (equal), writing – review and editing (supporting). **Yoshitsugu Mitani:** data curation (equal), writing – review and editing (supporting). **Adel K. El‐Naggar:** data curation (equal), writing – review and editing (supporting). **Renata Ferrarotto:** conceptualization (lead), data curation (supporting), project administration (lead), resources (lead), supervision (lead), visualization (lead), writing – review and editing (lead).

## Ethics Statement

This study was conducted in accordance with the Declaration of Helsinki. The study was approved by the Institutional Review Board of The University of Texas MD Anderson Cancer Center (PA‐17‐0865), which waived the requirement for informed consent because this was a retrospective study and all patients are deceased.

## Conflicts of Interest

Renata Ferrarotto reports personal fees from Regeneron, Sanofi, Merck Serono, Elevar Therapeutics, Prelude Therapeutics, Eisai Inc., Remix Therapeutics, Coherus BioSciences, and Bicara Tehrapeutics and nonfinancial support from Ayala Pharmaceuticals, EMD Serono, ISA, Genentech/Roche, Merck Serono, Pfizer, Viracta, and Gilead outside the submitted work. The other authors declare no conflicts of interest.

## Supporting information


Figures S1‐S5

## Data Availability

The datasets used and/or analyzed during the current study are available from the corresponding author upon reasonable request.
