# Supplementary material for: Clinical Outcomes With Notch Inhibitors in Notch‐Activated Recurrent/Metastatic Adenoid Cystic Carcinoma
Source: Cancer Med. 2025 Mar 2;14(5):e70663. doi: 10.1002/cam4.70663 (PMC11872804; doi:10.1002/cam4.70663)
Supplement: Supplementary file 1 — Figures S1‐S5 [file CAM4-14-e70663-s001.docx]

**Supplementary Material**

CLINICAL OUTCOMES WITH NOTCH INHIBITORS IN NOTCH-ACTIVATED RECURRENT/METASTATIC ADENOID CYSTIC CARCINOMA

**Supplementary Figure 1. For 20 patients, location and type of *NOTCH1* mutation was known, as annotated below.**

**
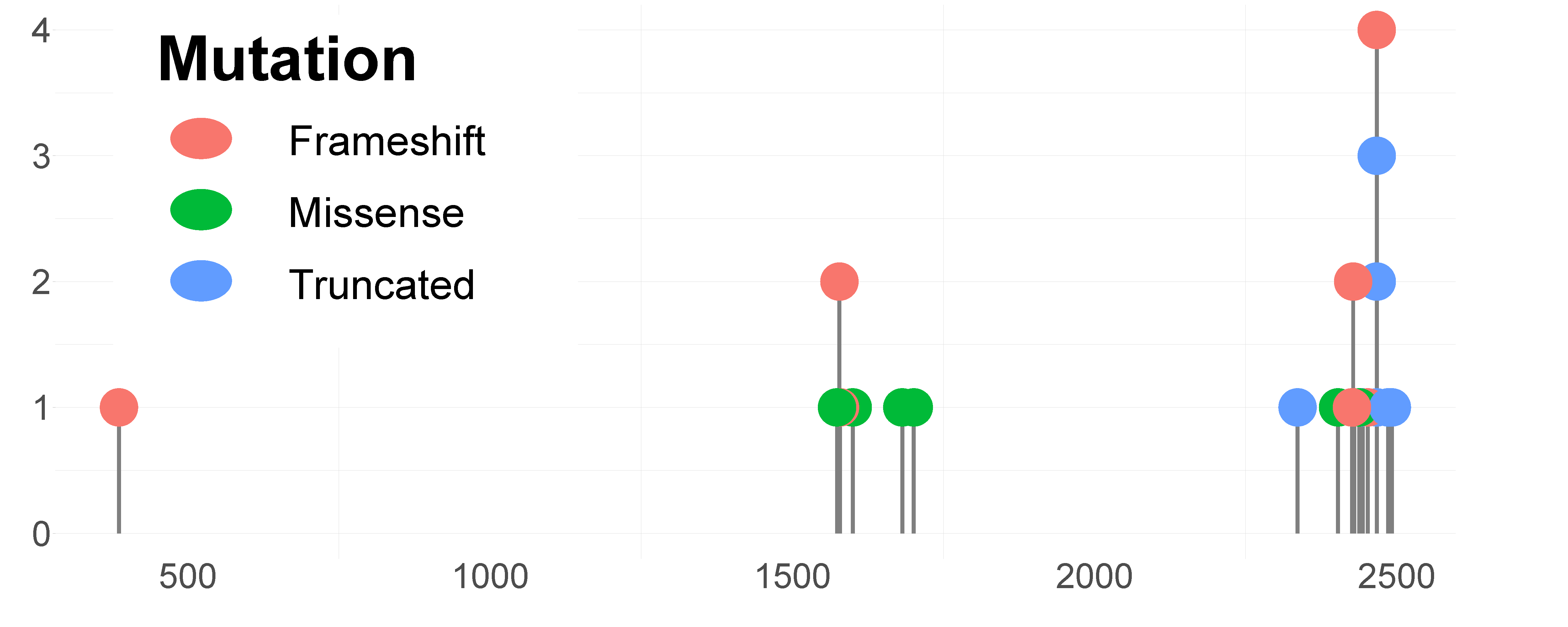
**

PEST Domain

NRR Domain

EGF Domain

**Supplementary Figure 2. Overall survival (OS) analysis comparing a) Solid/Basaloid vs. Non-Solid histology and b) *NOTCH*-mutant vs. *NOTCH*-non-mutant.**

2b.

2a.

**Supplementary Figure 3. Comparison of duration of response (DOR) between AL101 responders vs. Brontictuzumab responders.**

**Supplementary Figure 4. Comparison of progression-free survival (PFS) between NOTCH-mutants with mutations in the PEST domain vs. NRR domain.**

**Supplementary Figure 5. Circulating tumor DNA (ctDNA) during treatment with AL101 with detectable *NOTCH1* mutational variant.** Patient ctDNA followed ACC disease burden with initial radiological tumor shrinkage (SD per RECIST), followed by PD during AL101 therapy.


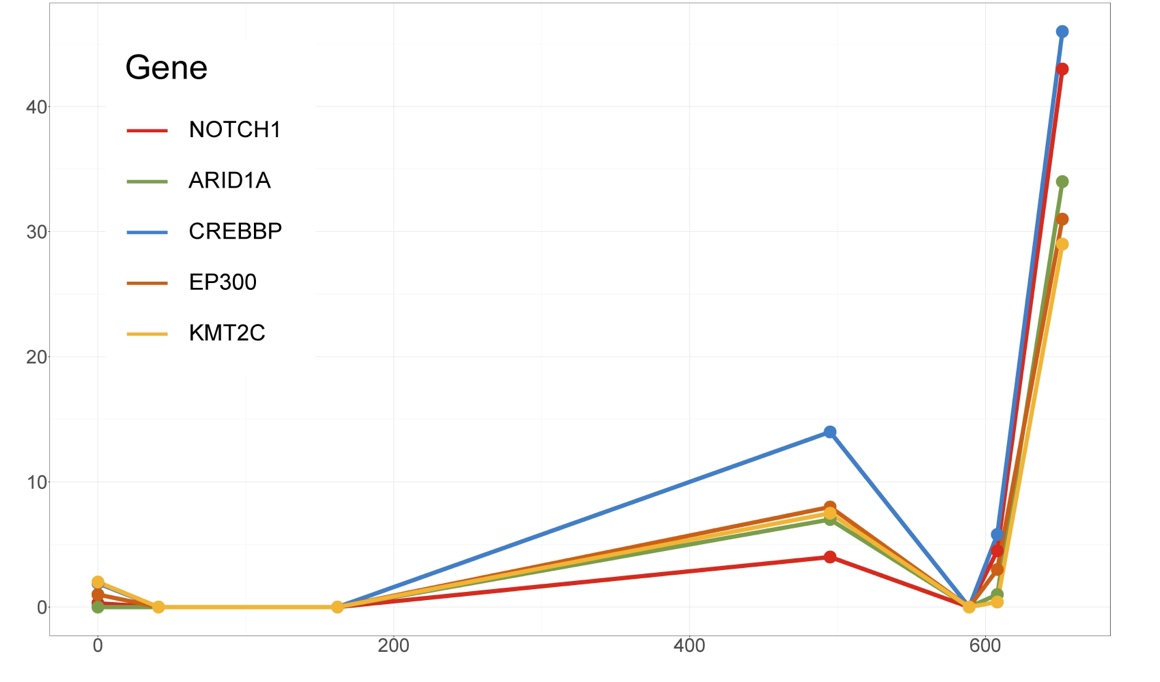


Days

ctDNA in Plasma

Enrollment in AL101 Trial

Diagnosis

Post-surgery and CRT

Local Recurrence

**SD**

**PD**
